# Supplementary material for: Theoretical Analysis for Using Pulsed Heating Power in Magnetic Hyperthermia Therapy of Breast Cancer
Source: Int J Mol Sci. 2021 Aug 18;22(16):8895. doi: 10.3390/ijms22168895 (PMC8396204; doi:10.3390/ijms22168895)
Supplement: Supplementary file 1 [file ijms-22-08895-s001.zip › ijms-1331398-supplementary.pdf]

Supplementary Material

Theoretical Analysis for Using Pulsed Heating Power in Magnetic Hyperthermia Therapy of Breast Cancer

Thanh-Luu Cao[1], Tuan-Anh Le[1,2], Yaser Hadadian[1] and Jungwon Yoon[1]*

[1] Gwangju Institute of Science and Technology, School of Integrated Technology, 123 Cheomdangwagiro, Buk-gu, Gwangju 61005, Korea; caothanhluu1988@gm. gist.ac.kr (T.L.C.); yaser.hadadian@gmail.com (Y.H.)
[2] Thuyloi University, 175 Tay Son, Dong Da, Ha Noi, Viet Nam. (e-mail: tuananhle@gm.gist.ac.kr or letuananh@tlu.edu.vn (T.A.L))
*Correspondence: jyoon@gist.ac.kr; Tel.: +82-62-715-5332

Table S1. Fraction of tissues damage at tumor domain at various source diameters for the pulsed and continuous powers for 30-minute simulation.

duty	Cycle	Source diameter (mm)					
	(second)	1.5	2	3	4	5	6
	5	0.93525	0.95048	0.9636	0.97737	0.93534	0.92093
	10	0.93532	0.95027	0.96355	0.97731	0.93524	0.921
	15	0.9353	0.95026	0.96345	0.97726	0.93516	0.92092
	20	0.93528	0.95025	0.96337	0.9772	0.93511	0.92083
	25	0.93526	0.95021	0.96339	0.97714	0.93504	0.92076
	30	0.93533	0.95022	0.96335	0.97713	0.93498	0.92071
	35	0.93532	0.95023	0.9633	0.97713	0.93494	0.92065
	40	0.93537	0.95021	0.96329	0.97715	0.93489	0.92063
0.9	45	0.93539	0.9502	0.9632	0.97715	0.93489	0.92067
	50	0.93518	0.95021	0.9633	0.97713	0.93484	0.92062
	55	0.93529	0.95026	0.96338	0.97711	0.93494	0.92055
	60	0.93554	0.9503	0.9632	0.97708	0.93502	0.92044
	65	0.93527	0.95018	0.96316	0.97702	0.93498	0.92042
	70	0.93535	0.94992	0.96315	0.97696	0.93486	0.92027
	75	0.93525	0.95008	0.96311	0.97694	0.9347	0.92036
	80	0.93497	0.94981	0.96312	0.97685	0.93471	0.92031
	85	0.9346	0.94961	0.96293	0.97675	0.93444	0.92024
	90	0.93489	0.94959	0.9627	0.97655	0.93389	0.91994
	95	0.93453	0.94935	0.96257	0.97635	0.93362	0.91917
1.0	100	**0.98687**	**0.99241**	**0.99607**	**0.99864**	**0.98692**	**0.9808**

Table S2. Fraction of tissues damage at tumor neighboring domain at various source diameters for the pulsed and continuous powers for 30-minute simulation.

duty	Cycle (second)	Source diameter (mm)					
		**1.5**	**2**	**3**	**4**	**5**	**6**
	5	0.07503	0.085395	0.097147	0.11444	0.076115	0.068896
	10	0.07509	0.085246	0.097136	0.11438	0.076076	0.068956
	15	0.075094	0.085271	0.097085	0.11439	0.076064	0.068948
	20	0.075119	0.085285	0.096976	0.11437	0.076073	0.068937
	25	0.075136	0.085304	0.097073	0.11436	0.076074	0.068937
	30	0.075175	0.085328	0.097083	0.11437	0.076083	0.068952
	35	0.07521	0.085345	0.097065	0.1144	0.076089	0.068945
	40	0.075227	0.085379	0.097067	0.11443	0.076111	0.068987
0.9	45	0.075259	0.085387	0.097	0.1145	0.076154	0.069041
	50	0.075181	0.085432	0.097176	0.11451	0.076165	0.069059
	55	0.075309	0.08547	0.097271	0.11459	0.076269	0.069061
	60	0.075351	0.085538	0.097155	0.11459	0.076287	0.069086
	65	0.075398	0.085546	0.097164	0.11462	0.076289	0.069143
	70	0.075402	0.085537	0.09724	0.11459	0.076338	0.069175
	75	0.075318	0.085588	0.097298	0.11465	0.076294	0.069224
	80	0.075186	0.085721	0.097355	0.11464	0.076364	0.069251
	85	0.075097	0.085757	0.097265	0.11461	0.076362	0.069257
	90	0.075511	0.085763	0.097335	0.11462	0.076296	0.069203
	95	0.075426	0.085658	0.097382	0.1147	0.076306	0.069135
1.0	100	**0.13322**	**0.14981**	**0.16778**	**0.19286**	**0.13445**	**0.12248**
